# Solubility Enhancement of Active Pharmaceutical Ingredients through Liquid Hydrotrope Addition: A Thermodynamic Analysis

**DOI:** 10.1021/acs.molpharmaceut.4c01117

**Published:** 2025-02-13

**Authors:** Sahar Nasrallah, Mirjana Minceva

**Affiliations:** Biothermodynamics, TUM School of Life Sciences, Technical University of Munich, Maximus-von Imhof-Forum 2, Freising 85354, Germany

**Keywords:** drugs solubility, solid−liquid equilibria, ternary SLE, thermodynamic modeling, pharmaceutical formulation

## Abstract

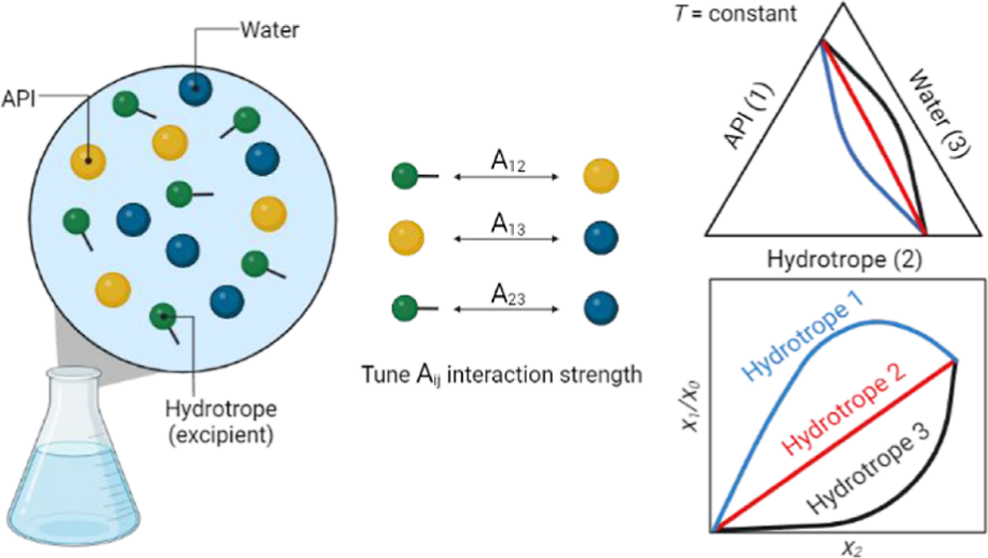

The poor water solubility of active pharmaceutical ingredients (APIs) poses a significant challenge in pharmaceutical development, affecting their bioavailability and therapeutic efficacy. Consequently, there is an urgent demand for strategies to improve API solubility, with hydrotropy emerging as one of the most effective approaches. Hydrotropes, which can act as excipients in pharmaceutical formulations, enhance solubility by solubilizing hydrophobic compounds in aqueous solutions through mechanisms other than micellar solubilization. However, identifying the right hydrotropic agent requires a screening from a large pool of candidates. This work aims to analyze hydrotropy from a thermodynamic perspective by investigating the influence of the molecular interactions among the API, hydrotrope, and water on the API solubility in water at different temperatures. For this systematic study, hypothetical ternary systems were used and only liquid hydrotropes were considered. Utilizing the Two-Suffix Margules equation to model the liquid phase nonideality, the study revealed that strong API–hydrotrope interactions notably enhance the API solubility in water. Additionally, the interaction between the hydrotrope and water significantly influences API solubility; weaker hydrotrope–water interactions allow for increased API solubility in water. However, when hydrotrope–water interactions are stronger than API–hydrotrope interactions, this effect is diminished. The theoretical findings were validated using solubility experimental data of syringic acid with alkanediols in water from the literature. The results of this work will aid in selecting suitable liquid hydrotropes for enhancing the API solubility in aqueous solutions.

## 1 Introduction

In the complex world of pharmaceutical development, the poor water solubility of active pharmaceutical ingredients (APIs) is a critical determinant that influences drug bioavailability and therapeutic efficacy. The biopharmaceutical classification system (BCS) acts as a navigational guide, classifying drugs into four categories (I to IV) based on their solubility and permeability characteristics.^1^ Statistics reveal that approximately 40% of the existing market APIs and about 90% of emerging APIs in research and clinical trials exhibit poor water solubility, falling into classes II (low solubility and high permeability) and IV (low solubility and low permeability) according to the BCS classification.^2,3^ The poor API solubility can lead to delayed distribution within the body or, in some instances, result in active ingredients being excreted without absorption.^4^ Among the different available methods for API solubility enhancement,^5,6^ hydrotropy is emerging as one of the most simple-to-apply and effective approaches.

The concept of “hydrotropy” was first introduced by German chemist Carl Neuberg in 1916.^7^ He defined hydrotropes (excipients) as amphiphilic compounds capable of increasing the solubility of hydrophobic substances in water by mechanisms other than micellar solubilization. These mechanisms include hydrotrope self-aggregation, hydrotropic destruction of water structures, and complexation between the hydrotrope and API.^8^ Amphiphilic compounds have hydrophilic (water-attracting) and hydrophobic (water-repelling) parts within the molecule. Examples of commonly used hydrotropes are sodium salts of short alkylbenzenesulfonates, sodium salts of benzoates, aromatic fatty acids, urea, and nicotinamide.^7,9^ Recently, ionic liquids, deep eutectic solvents, and biobased solvents have been utilized as hydrotropes.^10−12^ Most of the conventionally used hydrotropes are inexpensive, stable, have low toxicity, and are environmentally friendly, which makes them suitable for pharmaceutical applications.^13^

While the hydrotropy concept is easy to apply in practice, selecting the most appropriate hydrotrope remains challenging due to the extensive variety of available compounds. This selection process requires a deep understanding of the hydrotropy mechanism, which has been debated for many years. Hydrotrope-solute interactions have been studied using various methods, such as NMR spectroscopy.^14,15^ The selection of hydrotropes has relied on trial-and-error experimental screening to identify those that exhibit strong API–hydrotrope interactions.^16^ However, when the ability of the hydrotrope to enhance API solubility in water is assessed, the interactions among API–hydrotrope, API–water, and hydrotrope–water should be considered. To the best of our knowledge, no study has comprehensively investigated the influence of all interactions among API, hydrotrope, and water on API solubility.

From the solid–liquid equilibrium (SLE) perspective, the solubility of an API in the ternary API/hydrotrope/water system depends on the melting properties of the pure components and their activity coefficients in the liquid phase. The activity coefficients of components in the liquid phase quantify the intermolecular interactions among the API, hydrotrope, and water. Thermodynamic models, such as activity coefficient models, can be used to describe the nonideality of the liquid solution and calculate the activity coefficients of the components in the liquid phase. Thermodynamic models are broadly classified as correlative, predictive, or semipredictive. Correlative models, such as Van Laar,^17^ Margules,^18^ non-random two-liquid (NRTL),^19^ and UNIQUAC,^20^ utilize experimental equilibrium data to fit adjustable parameters and calculate solubility without explicitly relying on the molecular structure of the components. Predictive models, including Hansen,^21^ UNIFAC,^22^ and COSMO-RS,^23^ calculate the activity coefficients to estimate solubility based on the molecular structure and theoretical principles, thereby reducing reliance on experimental data. Semipredictive models, such as PC-SAFT^24^ and NRTL-SAC,^25,26^ integrate aspects of both approaches by combining structural information with limited experimental input.

This work investigates the enhancement of the API solubility in water by the addition of a hydrotrope that is liquid at the temperature of the solution, assuming hypothetical ternary systems composed of a model API, a model hydrotrope, and water. The scenario with solid excipients, including hydrotropes, has been discussed in our previous publication.^27^ For the systematic study in this work, a hypothetical ternary system composed of an API, hydrotrope, and water was considered. The study focuses on understanding how the nonideality of the liquid phase influences the solubility enhancement of the API from a thermodynamic perspective. We considered all pairwise molecular interactions within the system and analyzed the effect of temperature on the solubility of the API. The Two-Suffix Margules equation was used to model the nonideality of the hypothetical ternary liquid solution. Theoretical analysis was validated using data from the literature,^16^ applying the NRTL model,^19^ which enabled the analysis of binary interaction and self-component energies in the system. This study aims to highlight the role of thermodynamic modeling in guiding the selection of suitable liquid hydrotropes and their concentrations in enhancing API solubility in water.

## 2 Methods

### 2.1 Solid–Liquid Equilibrium

**Figure 1:**
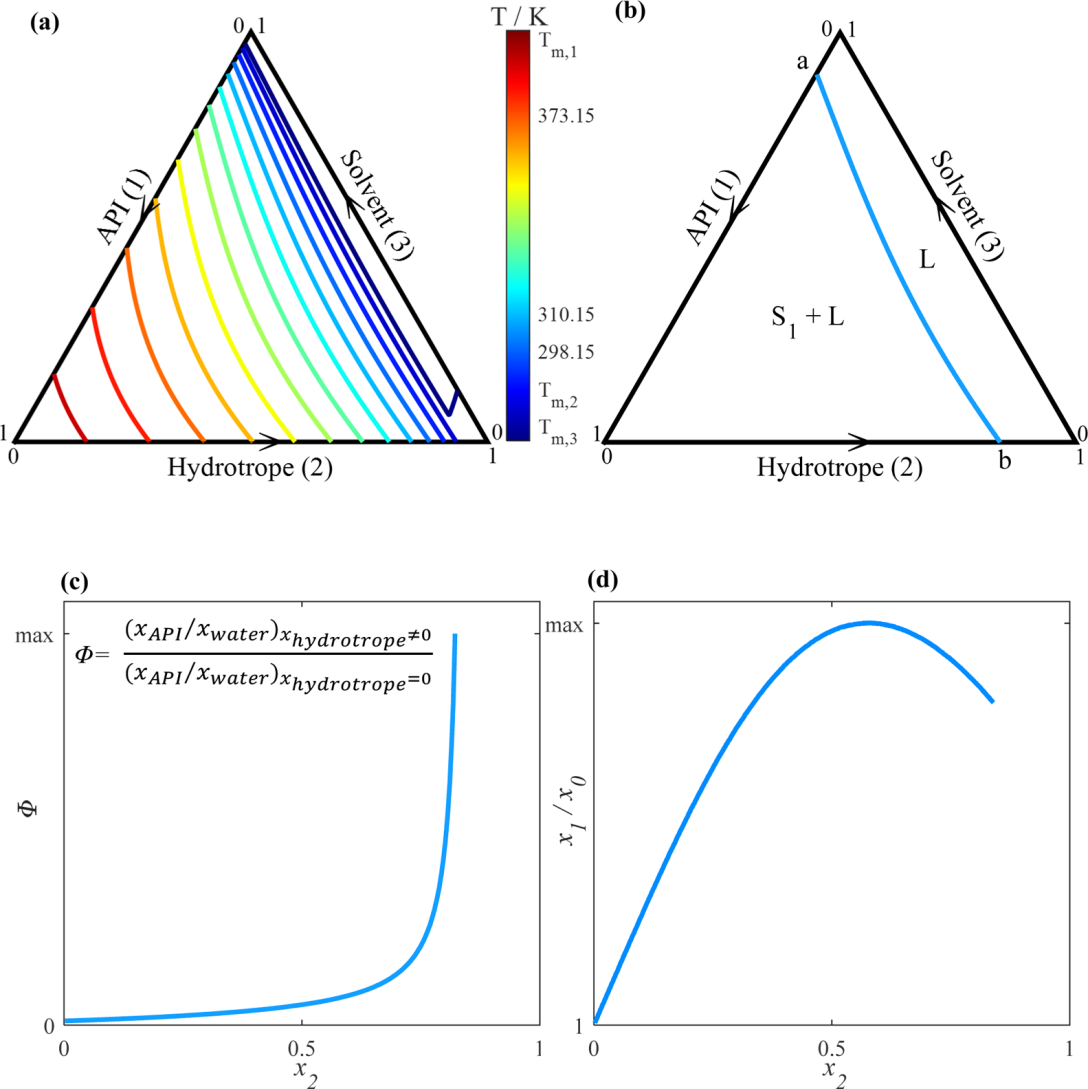
(a) SLE diagram of a hypothetical ternary API (1)/hydrotrope (2)/water (3) system. The lines represent the solubility isotherms calculated using the Two-Suffix Margules model at different temperatures. (b) Solubility line of the API in the ternary API (1)/hydrotrope (2)/water (3) system at a constant temperature. (c) Schematic representation of the API solubility enhancement factor (Φ) at a constant temperature and different hydrotrope mole fractions (*x*_2_). (d) Schematic representation of the API mole fraction in the ternary mixture (*x*_1_) over API mole fraction in pure water (*x*_0_) at a constant temperature and different hydrotrope mole fractions (*x*_2_).

SLE data can be represented graphically by constructing a phase diagram. Figure 1a illustrates the solubility isotherms of the ternary system consisting of API (1), hydrotrope (2), and water (3) at different temperatures. In specific temperature ranges lower than the melting temperature of the hydrotrope, where the hydrotrope is solid, ternary mixtures exhibit a eutectic point “eu”, where the solubility lines of the API and hydrotrope intersect. At higher temperatures, where the hydrotrope is in its liquid state, there is no hydrotrope solubility line in the ternary phase diagram. The isotherms represent only the API solubility lines, indicating that the hydrotrope is completely miscible in aqueous solution. Figure 1b shows a single solubility isotherm without a eutectic point (liquid hydrotrope) and the phases formed in each region of the phase diagram. At the solubility isotherm, solid API (S_1_) is in equilibrium with the liquid phase (L), which consists of API, hydrotrope, and water. The API solubility line extends between points ″a″ and ″b″. These solubility lines intersect the ternary phase diagram axis at the solubility of the API in the pure water (point ″a″) or the pure hydrotrope (point ″b″) at the temperature corresponding to the solubility line. This study exclusively focuses on liquid hydrotropes at the solution temperature, and thus, the presented solubility isotherms in the following sections include only the API solubility line.

### 2.2 Modeling of Solid–Liquid Equilibria

The solubility line of the API in Figure 1b can be calculated using the following equation^28^
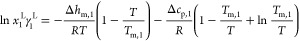
1where *x*_1_^L^ and γ_1_^L^ are the mole fraction and the activity coefficient of the API in the liquid phase, respectively; *T* is the temperature; Δ*h*_m_ and *T*_m_ are the melting enthalpy and temperature of pure API, respectively; Δ*c*_p_ is the difference between the constant pressure heat capacity of pure API in the solid and liquid states at *T*_m_; and *R* is the universal gas constant. In many cases, the Δ*c*_p_ term (second term on the right-hand side) has a smaller influence on the solubility curve compared to the Δ*h*_m_ term (first term on the right-hand side).^28^ Thus, for the sake of simplicity, the Δ*c*_p_ term was not considered in this study, and the following expression was used to calculate the solubility line
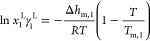
2

The activity coefficients of the components in the liquid phase were calculated using the Two-Suffix Margules activity model as follows^28^
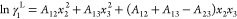
3

Expressions for γ_2_^L^ and γ_3_^L^ are similar to those for γ_1_^L^ and can be derived by swapping the indexes. To obtain γ_2_^L^, index 1 is replaced with 2, 2 with 3, and 3 with 1. To obtain γ_3_^L^, index 1 is replaced with 3, 3 with 2, and 2 with 1. In the ternary system API (1)/hydrotrope (2)/water (3), the parameters *A*_12_, *A*_23_, and *A*_13_ represent the binary interaction parameters between the components.

In addition to the Two-Suffix Margules activity model, the NRTL model was applied to calculate the binary interaction energy parameters (*U*) to determine the self-component binary energy interactions^19^
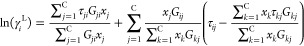
4
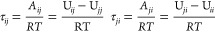
5
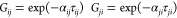
6where *A*_*ij*_ and *A*_*ji*_ are the binary interaction parameters between components *i* and *j*; *x*_*i*_ is the mole fraction of component *i*; and α_*ij*_ denotes the nonrandomness factor. If *A*_*ij*_ is assumed to equal *A*_*ji*_ and the mixture is assumed to be completely random (α_*ij*_ = 0), the NRTL model can be simplified to the Two-Suffix Margules activity model.^28^ The parameters *U*_*ij*_, *U*_*ji*_, *U*_*ii*_, and *U*_*jj*_ denote the binary interaction energy parameters between the molecules in the system, including self-interactions. The value of *U*_*ij*_ is always equal to that of *U*_*ji*_, indicating symmetry in the interaction energies between pairs of molecules. However, *A*_*ij*_ is not necessarily equal to *A*_*ji*_. For the ternary system, the binary interaction parameters are defined as follows
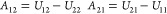
7
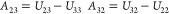
8
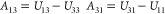
9

The NRTL model parameters were obtained by fitting experimental phase equilibria data of the respective systems using the following objective function
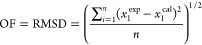
10where RMSD is the root-mean-square deviation, measuring the discrepancy between the experimentally measured mole fraction *x*_1_^exp^ and the calculated mole fraction *x*_1_^cal^ for *n* data points.

### 2.3 Solubility Enhancement Factor

In this study, solubility (*S*) was defined as the moles of the API dissolved in moles of water at a specific temperature and calculated using the following equation
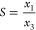
11where *x*_1_ and *x*_3_ are the mole fraction of API and water, respectively. The API solubility enhancement due to the addition of the hydrotrope was evaluated using the solubility enhancement factor (Φ), which was defined as the ratio between the API solubility in the presence of the hydrotrope and its solubility in the absence of the hydrotrope^29^
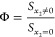
12where *x*_2_ is the hydrotrope mole fraction. According to eqs 11 and 12, Φ increases with the increasing API mole fraction (*x*_1_) or with the decreasing water mole fraction (*x*_3_) in the solution. Figure 1c shows an example of how Φ changes with the change of the hydrotrope mole fraction (*x*_2_) at a constant temperature along the solubility isotherm. As (*x*_3_) approaches 0, Φ becomes infinity. Figure 1d illustrates the variation in API mole fraction (*x*_1_) along the API solubility line relative to the API solubility in pure water (*x*_0_) (equivalent to point ″a″ in Figure 1b) at constant temperature.

In this study, the figures illustrating the variation of (*x*_1_/*x*_0_) with the hydrotrope mole fractions were used to show and discuss how API solubility is influenced by the intermolecular interactions between the components in the liquid solution at a specific temperature. The representations with Φ were employed to display the variation of the API solubility enhancement factor with the hydrotrope mole fraction at different temperatures. The Φ presented in this work was calculated at the point, where the hydrotrope mole fraction is 0.5 (*x*_2_ = 0.5). This point provides a balanced representation of the effect of the hydrotrope on API solubility, avoiding extreme values, where Φ might become impractically high or low. It also allows for consistent comparisons across different hydrotropes and conditions.

### 2.4 Melting Properties of Pure Components

The melting properties of a pure component are influenced by molecular symmetry, conformational diversity, and intermolecular forces.^30^ At the melting point, the melting properties are interrelated by the following equation
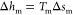
13where Δ*s*_m_ is the melting entropy.

The selected melting properties are typical for APIs and liquid hydrotropes, which have dissimilar molecular structures. APIs are generally rigid molecules with ordered crystalline structures, while hydrotropes exhibit less ordered crystal structures. To represent an API with low solubility in water, a high melting temperature (400 K) and entropy (Walden’s rule; Δ*s*_m_ = 54.4 J mol^–1^ K^–1^)^31^ were assumed for the API; the melting enthalpy of the API was calculated with eq 13 (see Table 1). The definition of Walden’s rule in this study applies to ordered crystals of rigid molecules.^31^ The hydrotrope was assumed to have a melting entropy of Δ*s*_m_ = 28.1 J mol^–1^ K^–1^ to represent components with high solubility in water.^31^ The hydrotrope melting temperature of 285 K was assumed to simulate liquid hydrotropes at the solution temperature and atmospheric pressure, with the melting enthalpy calculated using eq 13.

**Table 1:** Melting Properties of the API, Hydrotrope, and Water Used for Solid–Liquid Equilibrium Modeling

component	*T*_m_/K	Δ*s*_m_/J mol^–1^ K^–1^	Δ*h*_m_/k J mol^–1^
API (1)	400	54.4	21.8
hydrotrope (2)	285	28.1	8.0
water (3)	273^a^	22.0	6.01^a^

a Taken from ref (32).

## 3 Results and Discussion

The Results section is divided into two main subsections. In Section 3.1, the results of a systematic study of the intermolecular interactions’ influence on the API solubility determined with the Two-Suffix Margules model for a hypothetical system are presented. The range in which the binary interaction parameters were varied was selected to ensure a significant and observable change in the API solubility. To validate the theoretical findings from Section 3.1, the NRTL activity model was utilized with experimental data from literature,^16^ allowing for a comprehensive consideration of all molecular interactions in Section 3.2.

### 3.1 Theoretical Analysis of the Intermolecular Interactions’ Influence on the API Solubility

As mentioned in the previous section, the API solubility lines vary depending on the molecular interactions among the API, hydrotrope, and water in the liquid solution. Consequently, the position of the maximum API solubility point in the ternary phase diagram also varies. Selecting different hydrotropes to enhance API solubility influences the behavior of the API solubility lines, where the API mole fraction and the API solubility enhancement factor can either increase or decrease to different extents with the hydrotrope mole fraction. This section presents a detailed analysis of the impact of the liquid phase nonideality on the behavior of API solubility lines in the ternary phase diagrams along with the resultant API solubility enhancement factors. The nonideality of the solution was considered to investigate the influence of the interactions among API–water (*A*_13_), hydrotrope–water (*A*_23_), and API–hydrotrope (*A*_12_) on the solubility of API. For this study, a temperature range from 300 to 325 K was selected, as this is a commonly used range in the pharmaceutical industry and includes the body temperature range. In this section, the values of the binary interaction parameters were varied to account for different scenarios, covering cases with positive and negative deviations from the ideal solution behavior. A summary of the binary interaction parameters (*A*_*ij*_) used in Sections 3.1.1 to 3.1.3 is provided in Table S1.

#### 3.1.1 Effect of API–Water Molecular Interactions (*A*_13_) on the API Solubility Enhancement

**Figure 2:**
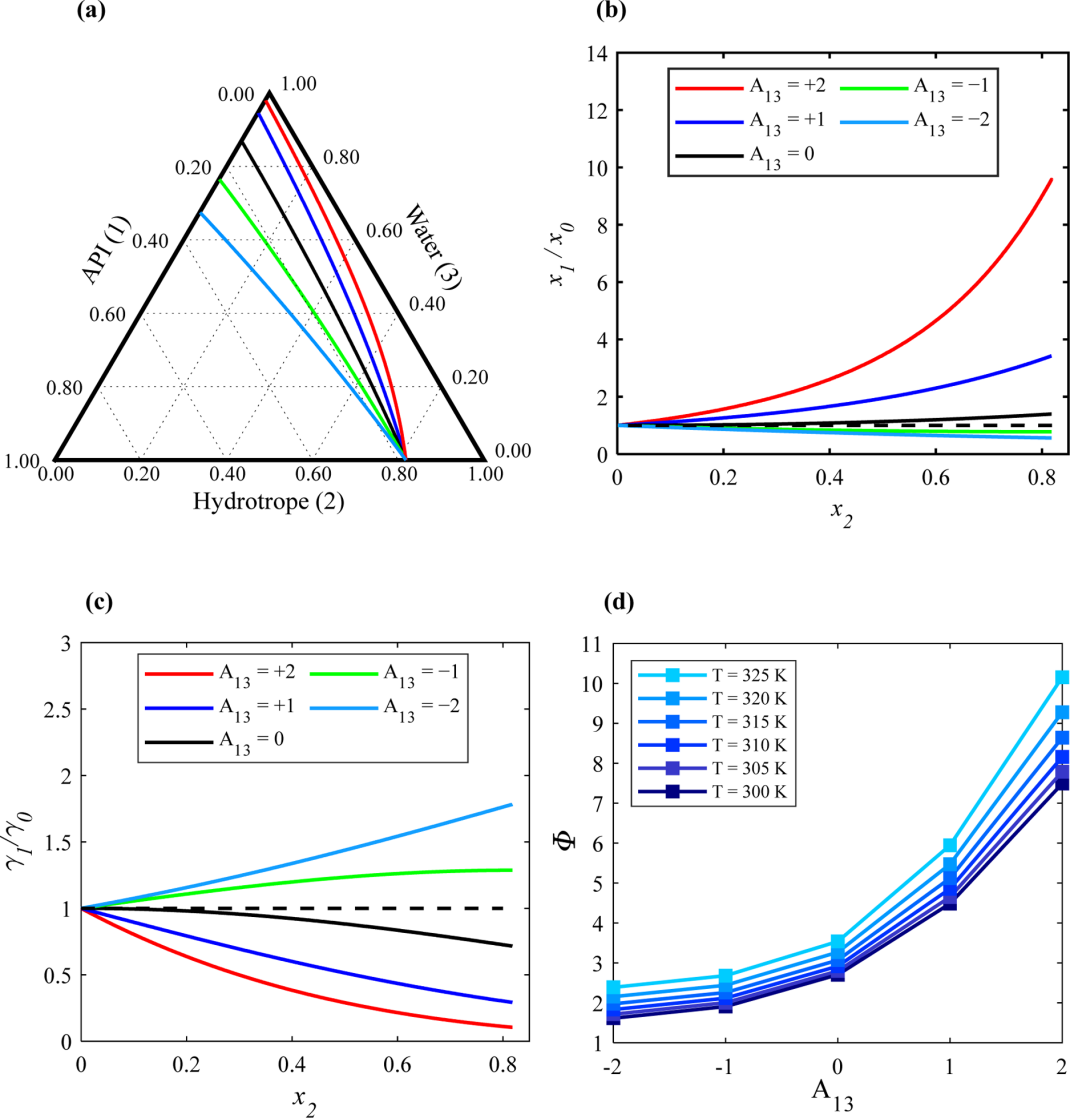
(a) SLE phase diagram of the API (1)/hydrotrope (2)/water (3) system at 305 K assuming different values of the API–water binary interaction parameter (*A*_13_) and *A*_12_ = −0.5 and *A*_23_ = −0.5, calculated using the Two-Suffix Margules model. (b) Corresponding (*x*_1_/*x*_0_) value calculated along the solubility isotherm at 305 K for different *A*_13_ values. (c) Corresponding (γ_1_/γ_0_) value calculated along the solubility isotherm at 305 K for different *A*_13_ values. (d) API solubility enhancement factor (Φ) at (*x*_2_ = 0.5) for different solution temperatures and varying values of the API–water binary interaction parameter (*A*_13_) and *A*_12_ = −0.5 and *A*_23_ = −0.5.

At first, the impact of API–water interactions (*A*_13_) on the API solubility was explored. Figure 2a shows the solubility isotherms of the ternary API (1)/hydrotrope (2)/water (3) system at 305 K, varying *A*_13_ from +2 to −2. The binary interaction parameters *A*_12_ and *A*_23_ were assumed to be −0.5, indicating favored interactions between the API–hydrotrope and hydrotrope–water. As depicted in Figure 2a, the API mole fraction changes along the API solubility lines, starting from the API solubility in pure water (equivalent to point ″a″ in Figure 1b). As the hydrotrope mole fraction increases in the liquid solution, the API solubility either rises or decreases until it reaches its solubility in the liquid hydrotrope (equivalent to point ″b″ in Figure 1b). The starting points (equivalent to ″a″) change with different *A*_13_ values, indicating that the API solubility in pure water varies. However, the end points remain constant, as the API–hydrotrope interactions are unchanged. The behavior of the API solubility lines changes with variations in the *A*_13_ interaction parameter. Figure 2b shows the corresponding (*x*_1_/*x*_0_) values along the solubility isotherm at 305 K for the same *A*_13_ values.

As observed in Figure 2b, if the API–water interactions are significantly stronger compared to API–hydrotrope and hydrotrope–water interactions (green and light blue lines), (*x*_1_/*x*_0_) is less than 1 and decreases as the hydrotrope mole fraction (*x*_2_) in the liquid solution increases, indicating that adding hydrotrope does not enhance API solubility in the liquid solution.

Conversely, when API–water interactions are less favored (black, blue, and red lines), (*x*_1_/*x*_0_) increases with increasing (*x*_2_) and exceeds 1, signifying that the API solubility in the solution increases with the addition of the hydrotropes. Figure 2c depicts how API activity coefficients in the liquid solution change relative to those in pure water, i.e., γ_1_/γ_0_ along the solubility isotherm at 305 K, varying with different *A*_13_ values. When API–water interactions are less favored compared to API–hydrotrope and hydrotrope–water interactions, (γ_1_/γ_0_) decreases as the hydrotrope mole fraction (*x*_2_) in the liquid solution increases. Conversely, when API–water interactions are more favored than hydrotrope–water and API–hydrotrope interactions (green and light blue lines), (γ_1_/γ_0_) increases with the increasing hydrotrope mole fraction. The SLE equation eq 2 demonstrates that as the activity coefficient of the API in the liquid solution (γ_1_) increases, the mole fraction of the API in the liquid phase (*x*_1_) decreases and vice versa.

Figure 2d illustrates the influence of changing the solution temperature on Φ for *x*_2_ = 0.5, assuming different *A*_13_ values and *A*_12_ = −0.5 and *A*_23_ = −0.5. It is evident that the temperature effect on Φ becomes more pronounced as the *A*_13_ values increase. For instance, with *A*_13_ = +2, Φ rises from approximately 7 to 10 when the temperature increases from 300 to 325 K. This suggests that the less favorable the API-water interactions, the more sensitive the API solubility is to temperature changes. On the other hand, for *A*_13_ < – 1, Φ shows only a slight increase with rising temperature. The SLE diagrams of the ternary system calculated at different temperatures and assuming different *A*_13_ values are provided in Figure S1 in the Supporting Information file.

#### 3.1.2 Effect of Hydrotrope–Water Molecular Interactions (*A*_23_) on the API Solubility Enhancement

**Figure 3:**
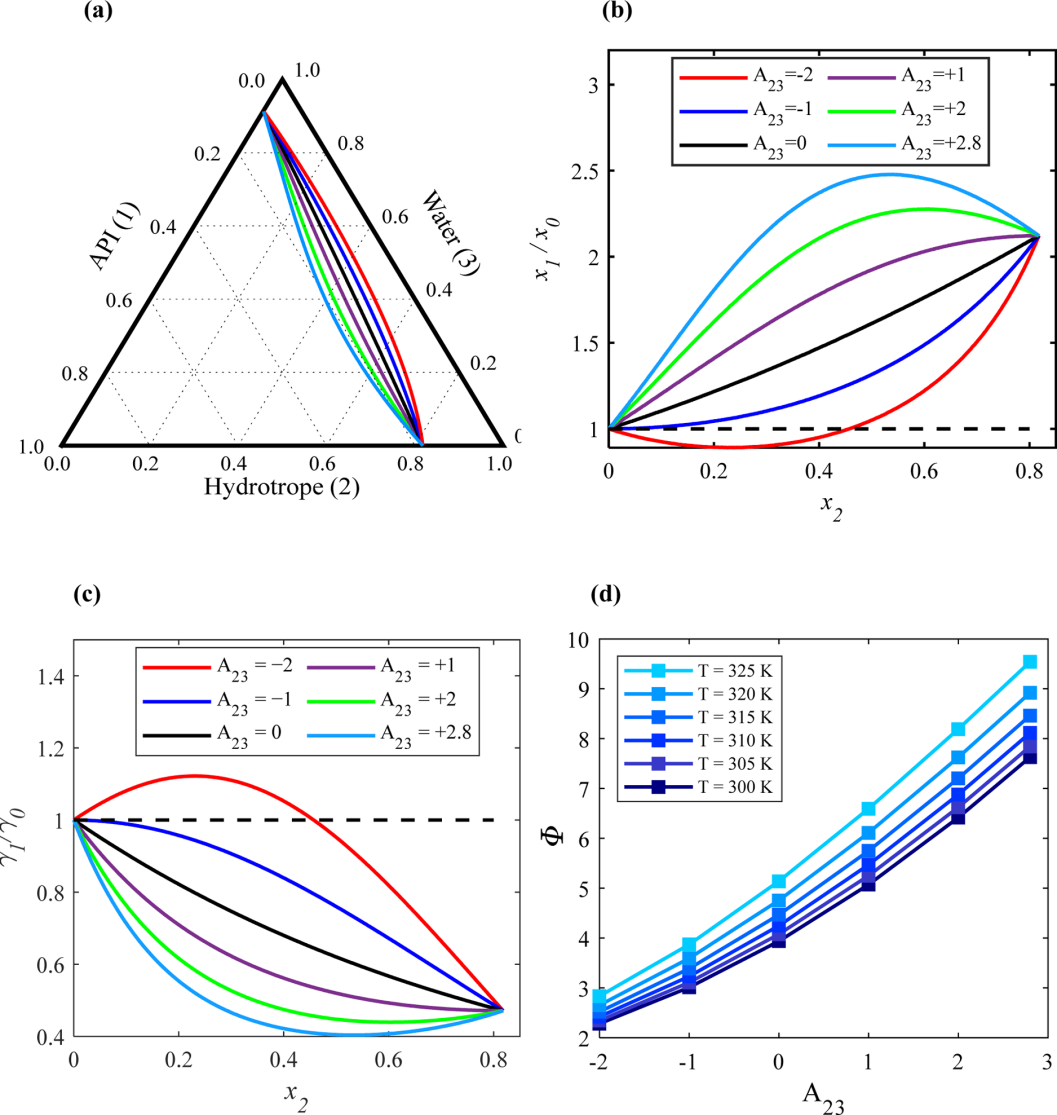
(a) SLE phase diagram of the API (1)/hydrotrope (2)/water (3) system at 305 K assuming different values of the hydrotrope–water binary interaction parameter (*A*_23_) and *A*_12_ = −0.5 and *A*_13_ = +0.5, calculated using the Two-Suffix Margules model. (b) Corresponding (*x*_1_/*x*_0_) value calculated along the solubility isotherm at 305 K for different *A*_23_ values. (c) Corresponding (γ_1_/γ_0_) value calculated along the solubility isotherm at 305 K for different *A*_23_ values. (d) API solubility enhancement factor (Φ) at (*x*_2_ = 0.5) for different solution temperatures and varying values of the hydrotrope–water binary interaction parameter (*A*_23_) and *A*_12_ = −0.5 and *A*_13_ = +0.5.

Next, the influence of hydrotrope–water interactions (the value of *A*_23_) on API solubility was investigated. Figure 3a shows the calculated solubility isotherms of the ternary system at 305 K when varying the *A*_23_ value between +2.8 and −2 and keeping *A*_13_ = +0.5 and *A*_12_ = −0.5, indicating unfavorable interactions between the API and water and favored interactions between the API and hydrotrope. The behavior of the API solubility lines between API solubility in pure water (equivalent to point ″a″ in Figure 1b) and API solubility in pure hydrotrope (equivalent to point ″b″ in Figure 1b) varies significantly with the strength of the hydrotrope–water interactions. Figure 3b,c shows the corresponding (*x*_1_/*x*_0_) and (γ_1_/γ_0_) values along the solubility isotherm at 305 K for various *A*_23_ values, respectively.

When the hydrotrope–water interactions are weaker and less favored than API–water interactions and API–hydrotrope interactions (green and light blue lines), (*x*_1_/*x*_0_) increases along the API solubility lines. This rise continues until the API solubility in the solution approaches its maximum. Beyond this point, (*x*_1_/*x*_0_) decreases to the solubility in pure hydrotrope (equivalent to point ″b″ in Figure 1b). This indicates that weaker hydrotrope–water interactions result in higher API solubility in the ternary mixture compared to API solubility in the pure hydrotrope.

The effect on solubility enhancement decreases as the hydrotrope–water interactions become slightly stronger but are still less favorable than the API–water and API–hydrotrope interactions (purple line).

The hydrotrope concentrations, where API mole fractions are highest in the ternary solution, correspond to those where API activity coefficients are lowest and vice versa. For weaker hydrotrope–water interactions compared to API–hydrotrope but stronger than API–water interactions (black line), (*x*_1_/*x*_0_) increases and (γ_1_/γ_0_) decreases nearly linearly with hydrotrope mole fraction.

Conversely, when the hydrotrope–water interactions are strong and more favored than the API–water and API–hydrotrope interactions (red and dark blue lines), (*x*_1_/*x*_0_) initially remains constant or decreases at low hydrotrope concentrations. However, with increasing hydrotrope concentration, (*x*_1_/*x*_0_) starts to increase again until it reaches the maximum API solubility in the liquid hydrotrope. The corresponding (γ_1_/γ_0_) values display a mirror image behavior to (*x*_1_/*x*_0_) values.

Figure 3d depicts the effect of varying the solution temperature on Φ when *x*_2_ = 0.5, considering various *A*_23_ values, with *A*_12_ = −0.5 and *A*_13_ = +0.5. It is evident that the temperature impact on Φ becomes more significant as the *A*_23_ values increase. For instance, when *A*_23_ = +2.8, Φ rises from approximately 7 to 10 as the temperature increases from 300 to 325 K. Conversely, for *A*_23_ < – 1, Φ only increases slightly from about 2.25 to 3 within the same temperature range. The SLE diagrams of the ternary system calculated at different temperatures and assuming different *A*_23_ values are reported in Figure S2 in the Supporting Information file.

#### 3.1.3 Effect of API–Hydrotrope Molecular Interactions (*A*_12_) on the API Solubility Enhancement

**Figure 4:**
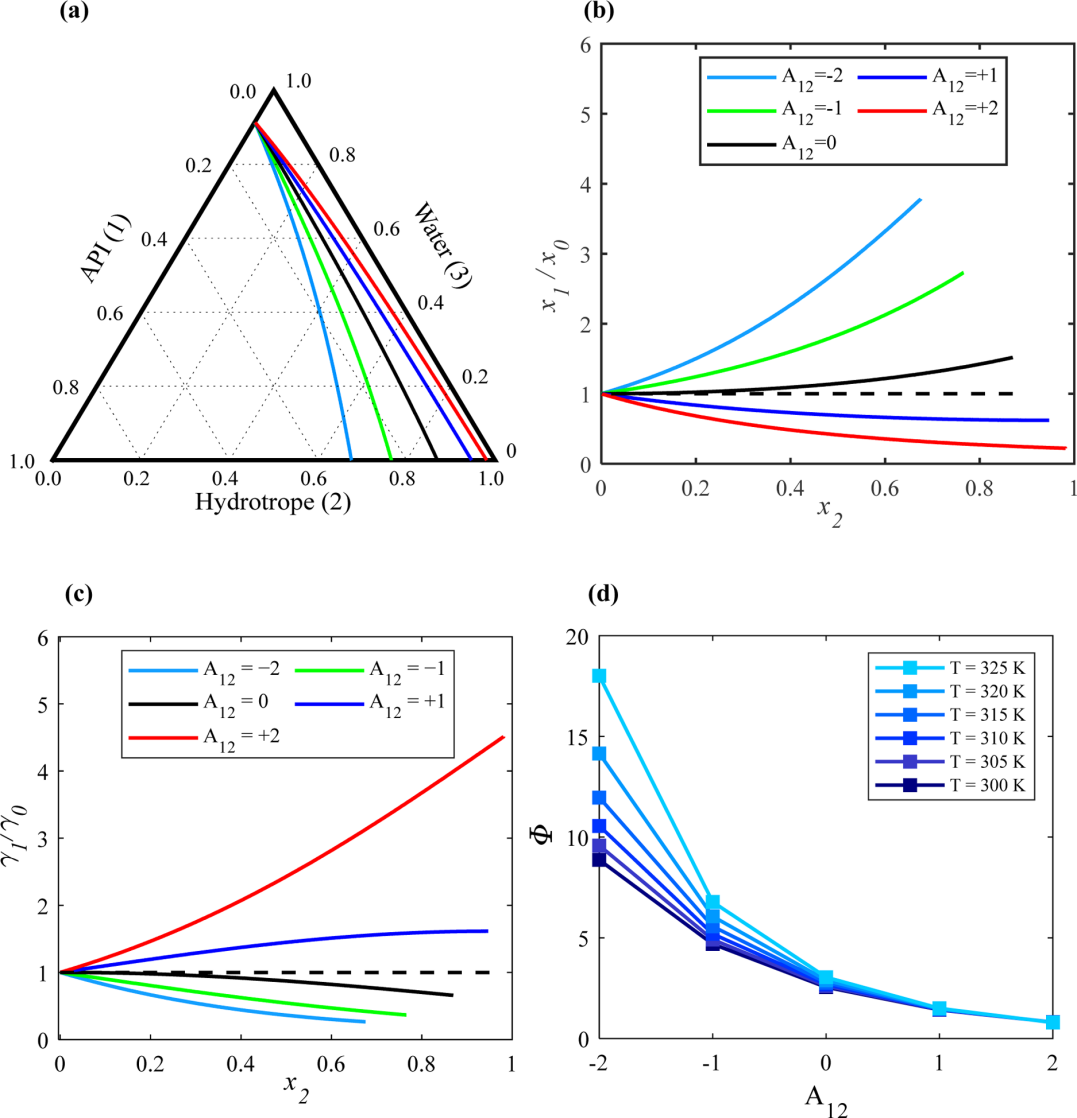
(a) SLE phase diagram of the API (1)/hydrotrope (2)/water (3) system at 305 K assuming different values of the API–hydrotrope binary interaction parameter (*A*_12_) and *A*_23_ = −0.5 and *A*_13_ = +0.5, calculated using the Two-Suffix Margules model. (b) Corresponding (*x*_1_/*x*_0_) values calculated along the solubility isotherm at 305 K for different *A*_12_ values. (c) Corresponding (γ_1_/γ_0_) values calculated along the solubility isotherm at 305 K for different *A*_12_ values. (d) API solubility enhancement factor (Φ) at (*x*_2_ = 0.5) at different solution temperatures and varying values of the API–hydrotrope binary interaction parameter (*A*_12_), and *A*_23_ = −0.5 and *A*_13_ = +0.5.

Lastly, the influence of the API–hydrotrope interactions (the value of *A*_12_) on the API solubility was explored. Figure 4a shows the calculated solubility isotherms of the ternary API (1)/hydrotrope (2)/water (3) system at 305 K when varying the value of *A*_12_ from +2 to −2 and assuming *A*_13_ = +0.5 and *A*_23_ = −0.5, indicating unfavorable interactions between the API and water and favored interactions between the hydrotrope and water. As depicted in Figure 4a, the API solubility lines begin from the same point since the API–water interactions are constant, but the end point varies with different API–hydrotrope interactions. Figure 4b,c displays the corresponding (*x*_1_/*x*_0_) and (γ_1_/γ_0_) values calculated along the solubility isotherms at 305 K obtained for different *A*_12_ values, respectively.

If the API–hydrotrope interactions are more favored than hydrotrope–water and API–water interactions (light blue and green lines), (*x*_1_/*x*_0_) increases as the mole fraction of the hydrotrope (*x*_2_) in the liquid solution increases. However, as the API–hydrotrope interactions become weaker (black line), the effect on solubility enhancement decreases. The corresponding (γ_1_/γ_0_) decreases as the hydrotrope mole fraction (*x*_2_) in the liquid solution increases.

In contrast, when API–hydrotrope interactions are unfavorable (the red and dark blue lines), the (*x*_1_/*x*_0_) value becomes less than 1, indicating that the API solubility in the liquid solution is lower than that in pure water. The corresponding (γ_1_/γ_0_) values increases as the hydrotrope mole fraction (*x*_2_) in the liquid solution increases.

Figure 4d illustrates the impact of changing the solution temperature on Φ at *x*_2_ = 0.5, considering different *A*_12_ values and *A*_23_ = −0.5 and *A*_13_ = +0.5. As seen in Figure 4d, the influence of temperature on Φ increases as the *A*_12_ value decreases. For instance, when the solution temperature increases from 300 to 325 K, Φ rises from around 8.8 to 18 when *A*_12_ = −2. Conversely, when *A*_12_ > 0, Φ slightly change with an increase in temperature from 300 to 325 K. Additionally, the temperature impact on Φ is significantly larger when changing the *A*_12_ compared to changing *A*_13_ and *A*_23_, as seen in Figures 2d and 3d, respectively. The SLE diagrams of the ternary system calculated at different temperatures and assuming that different *A*_12_ values are depicted in Figure S3 in the Supporting Information file.

### 3.2 Analysis of Molecular Interactions in Real Ternary Systems: a Case-Study of 1,2-Alkanediols

The theoretical analysis in the previous section demonstrated that API solubility in the ternary solution significantly increases as API–hydrotrope interactions strengthen. Conversely, if hydrotrope–water interactions exceed API–hydrotrope interactions, then API solubility decreases. This effect becomes more pronounced as API–water interactions become less favored. In this section, the theoretical findings on the impact of molecular interactions on API solubility in the ternary API (1)/hydrotrope (2)/water (3) system will be validated using experimental data from the literature.^16^ Syringic acid, known for its antioxidant, antimicrobial, anti-inflammatory, anticancer, and antidiabetic properties, represents API (1). The melting temperature and melting enthalpy of syringic acid are 482.5 K and 28.1 kJ mol^–1^, respectively,^33^ while melting entropy calculated using eq 13 is 58.2 J mol^–1^ K^–1^. In the work of Abranches et al.,^16^ the solubility of syringic acid was measured in water (3) in the presence of different 1,2-alkanediols as hydrotropes (2): 1,2-ethanediol, 1,2-propanediol, 1,2-butanediol, 1,2-pentanediol, and 1,2-hexanediol, at 303.2 K. The experimental data were converted into mole fractions, leading to differences in the shape and maximum points of the solubility lines to those in the original article. The molecular structures of syringic acid and the studied 1,2-alkanediols are provided in Table S2 of the Supporting Information file.

Table 2 presents the calculated interaction energy parameters (*U*) and the corresponding interaction parameters (*A*) using the NRTL model by fitting the experimental phase equilibria data, as described in (eqs 4–10). As shown in Table 2, increasing the alkyl chain length from 1,2-ethanediol to 1,2-pentanediol, (*U*_12_) becomes more negative, indicating a more favored interaction (more negative energy) between syringic acid and the hydrotrope. However, from 1,2-pentanediol to 1,2-hexanediol, there is a slight increase (less negative), indicating that the interaction strength stabilizes or slightly weakens at this point. The increasing hydrophobicity of the longer alkyl chains likely enhances the affinity of the hydrotrope for syringic acid due to stronger nonpolar interactions. However, this effect plateaus beyond a specific chain length (between pentanediol and hexanediol), possibly due to steric hindrance or solubility limitations.

**Table 2:** Calculated Binary Interaction Energy Parameters (*U*) and the Derived Binary Interaction Parameters (*A*) for Syringic Acid (1), Hydrotropes (2), and Water (3) in Ternary Systems with Different 1,2-Alkanediols at 303.2 K, Using the NRTL Activity Model^a^

	*U*_12_	*U*_23_	*U*_13_	*U*_11_	*U*_22_	*U*_33_	*A*_12_	*A*_21_	*A*_23_	*A*_32_	*A*_13_	*A*_31_	RMSD (%)
1,2-ethanediol	0.73	8.30	11.23	–3.95	3.92	13.68	–3.19	4.68	–5.39	4.37	–2.45	15.19	0.004
1,2-propanediol	–0.34	9.61	11.23	–3.95	2.57	13.68	–2.90	3.62	–4.07	7.05	–2.45	15.19	0.009
1,2-butanediol	–2.25	10.07	11.23	–3.95	–0.54	13.68	–1.71	1.71	–3.61	10.61	–2.45	15.19	0.019
1,2-pentanediol	–7.90	12.94	11.23	–3.95	–15.52	13.68	7.61	–3.95	–0.74	28.46	–2.45	15.19	0.023
1,2-hexanediol	–7.85	11.75	11.23	–3.95	–16.32	13.68	8.47	–3.89	–1.93	28.07	–2.45	15.19	0.015

a The units of *A* and *U* are kJ mol^–1^.

As the alkyl chain length increases, the (*U*_23_) values become more positive, indicating that hydrotrope–water interactions are less favored. This occurs because longer alkyl chains are more hydrophobic, making it more difficult for the hydrotrope to interact with water, a polar solvent. Interestingly, 1,2-pentanediol, slightly shorter, demonstrated the strongest water-repelling effect in the series. In contrast, the increase in the chain length of 1,2-hexanediol may allow the molecule to fold or orient in a way that maintains some level of interaction with water.

The self-interaction energy values of hydrotropes (*U*_22_) decrease from positive to negative as the alkyl chain length increases, indicating a transition from weaker to stronger self-interactions among the hydrotropes with the increasing alkyl chain length. Shorter chains exhibit positive values of *U*_22_ due to their lower hydrophobicity, leading to more interactions with the surrounding solvent. In contrast, longer chains become more hydrophobic, promoting stronger nonpolar interactions among hydrotrope molecules and resulting in negative (*U*_22_) values that reflect enhanced hydrotrope self-aggregation. A sharp increase in (*U*_22_) values for 1,2-pentanediol and 1,2-hexanediol, compared to shorter-chain 1,2-alkanediols, may be attributed to a threshold effect in hydrophobicity, where the longer alkyl chains lead to a significant increase in the self-aggregation of the hydrotrope molecules.

The interaction energy values between syringic acid and water (*U*_13_), the self-interaction energy of syringic acid (*U*_11_), and the self-interaction energy of water (*U*_33_) remain constant for all 1,2-alkanediols, as expected, since these two components do not change in the studied ternary systems.

**Figure 5:**
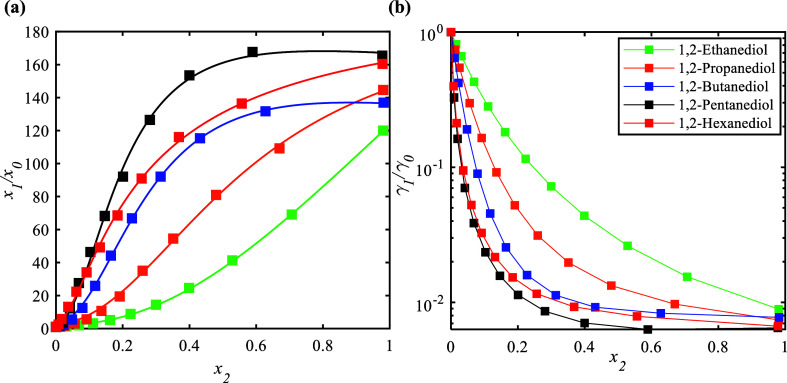
(a) Corresponding (*x*_1_/*x*_0_) values calculated along the solubility isotherm for syringic acid (1)/1,2-alkanediols (2)/water (3) system at 303.2 K. (b) Corresponding (γ_1_/γ_0_) values calculated along the solubility isotherm at 303.2 K for the same systems. In Figure 5a,b, the squares represent experimental data, while the lines were calculated with the NRTL model.

Figure 5a illustrates the corresponding (*x*_1_/*x*_0_) values calculated along the solubility isotherms at 303.2 K for all of the studied systems. In all cases, the (*x*_1_/*x*_0_) values rise as the mole fraction of the hydrotrope (*x*_2_) in the liquid solution increases. The selection of different 1,2-alkanediols significantly affects the solubility of syringic acid in water. Choosing 1,2-alkanediols with longer alkyl chain lengths results in a more significant enhancement of syringic acid solubility. This can be attributed to the more favored interaction energies between the hydrotrope and syringic acid and weaker interactions between the hydrotrope and water. For higher 1,2-alkanediols (1,2-pentanediol and 1,2-hexanediol), a smaller amount of hydrotrope is required to achieve an 80-fold enhancement in syringic acid solubility (*x*_1_/*x*_0_), approximately half of the maximum solubility enhancement observed in the experiments.

Figure 5b illustrates the syringic acid activity coefficients in the liquid solution relative to those in pure water (γ_1_/γ_0_) along the solubility isotherm at 303.2 K. The corresponding (γ_1_/γ_0_) values decrease as the hydrotrope mole fraction (*x*_2_) in the liquid solution increases. Furthermore, the (γ_1_/γ_0_) values decrease with an increase in the 1,2-alkanediol chain length, except for 1,2-hexanediol, as discussed previously. In addition to the syringic acid activity coefficients, the activity coefficients of 1,2-alkanediols and water in the ternary system were calculated, as shown in Figure S4a,b in the Supporting Information file. As the alkyl chain length increases, the (γ_2_/γ_0_) and (γ_3_/γ_0_) values rise from 1,2-ethanediol to 1,2-pentanediol, indicating decreased solubility of both the hydrotropes and water in the ternary system (higher activity coefficients), which corresponds to increasing solubility of syringic acid in the same order. However, 1,2-hexanediol deviates from this trend, exhibiting lower (γ_2_/γ_0_) and (γ_3_/γ_0_) values than those of the other hydrotropes, which could also explain the decreased syringic acid solubility. Note that syringic acid solubility follows the expected trend in the dilute range, but 1,2-hexanediol deviates as hydrotrope–water interactions become more dominant with the increasing hydrotrope concentration, reversing beyond (*x*_2_ = 0.1).

## 4 Conclusions

In this work, we employed thermodynamic modeling to understand the interplay of pairwise interactions among API (1), hydrotrope (2), and water (3), which plays a critical role in determining API solubility in water. These interactions influence the location of maximum solubility within the ternary system, helping to identify the most effective hydrotropes for enhancing API solubility. The impact of liquid phase nonideality and pairwise interactions on the solubility enhancement of the API in a hypothetical ternary API/hydrotrope/water system was investigated. It was found that more favored API–hydrotrope interactions compared to the API–water and hydrotrope–water interactions would significantly improve the API solubility in liquid solution. On the other hand, if the hydrotrope–water interactions are more favored than the API–hydrotrope interactions, the solubility of API in the solution would decrease. Thus, the best scenario for improving the solubility of API is to select a hydrotrope that strongly interacts with the API but moderately interacts with water. In Table 3, an overview of API solubility change with the addition of hydrotrope for different interaction strengths (weak, medium, or strong) between the API, hydrotrope, and water, based on the values of the binary interaction parameters (*A*_13_, *A*_12_, and *A*_23_) is provided. The theoretical findings were validated by using experimental solubility data for syringic acid in water with various 1,2-alkanediols from the literature, which confirmed our predictions about the impact of pairwise interactions on API solubility.

**Table 3:** Summary of the Qualitative Trends in API Solubility (*x*_1_/*x*_0_) with the Addition of the Hydrotrope (*x*_2_) under Different Scenarios of Binary Interaction Parameters, for API–Water Interactions (*A*_13_), API–Hydrotrope Interactions (*A*_12_), and Hydrotrope–Water Interactions (*A*_23_)

API–water interactions (*A*_13_)	API–hydrotrope interactions (*A*_12_)	hydrotrope–water interactions (*A*_23_)	API solubility trend
Section 3.1.1			
strong	medium	medium	decrease
medium	medium	medium	slightly increase
weak	medium	medium	significantly increase
Section 3.1.2			
weak	medium	very strong	decrease then increase
weak	medium	strong	slightly increase
weak	medium	weak	significantly increase^a^
Section 3.1.3			
weak	weak	medium	decrease
weak	strong	medium	slightly increase
weak	very strong	medium	significantly increase

a To a maximum (peak) and then slightly decrease.

The findings of this work aim to streamline pharmaceutical formulation development by minimizing the experimental effort required to identify effective hydrotrope candidates. The acquired knowledge of intermolecular interactions and their influence on API solubility, combined with thermodynamic models, enables the efficient screening and selection of hydrotropes to achieve targeted solubility. This approach supports the design of effective drug delivery systems and the development of novel API–hydrotrope combinations that enhance the solubility and bioavailability in water. The findings can also be extended to other solvents, which play important roles in API synthesis and purification (e.g., crystallization). Although this study focuses on theoretical modeling, further research is recommended to assess the safety of specific API–hydrotrope combinations and their optimal concentrations in biological systems.

## Supporting Information Available

SLE diagrams of the ternary system calculated at different temperatures and assuming different *A*_13_ values; SLE diagrams of the ternary system calculated at different temperatures and assuming different *A*_23_ values; SLE diagrams of the ternary system calculated at different temperatures and assuming different *A*_12_ values; activity coefficients of 1,2-alkanediols and water calculated along the solubility isotherm at 303.2 K in the ternary system; summary of the interaction parameters used for the ternary API (1)/hydrotrope (2)/water (3) system in different sections of the study; and molecular structures of syringic acid and the studied 1,2-alkanediols (PDF)
