# Supplementary material for: Solubility Enhancement of Active Pharmaceutical Ingredients through Liquid Hydrotrope Addition: A Thermodynamic Analysis
Source: Mol Pharm. 2025 Feb 13;22(3):1408–18. doi: 10.1021/acs.molpharmaceut.4c01117 (PMC11881036; doi:10.1021/acs.molpharmaceut.4c01117)
Supplement: Supplementary file 1 — mp4c01117_si_001.pdf [file mp4c01117_si_001.pdf]

Supporting Information

Solubility Enhancement of Active Pharmaceutical Ingredients through Liquid Hydrotrope Addition: A Thermodynamic Analysis

Sahar Nasrallah and Mirjana Minceva*

Biothermodynamics, TUM School of Life Sciences, Technical University of Munich, Maximus-von Imhof-Forum 2, Freising 85354, Germany

*Corresponding author e-mail: mirjana.minceva@tum.de

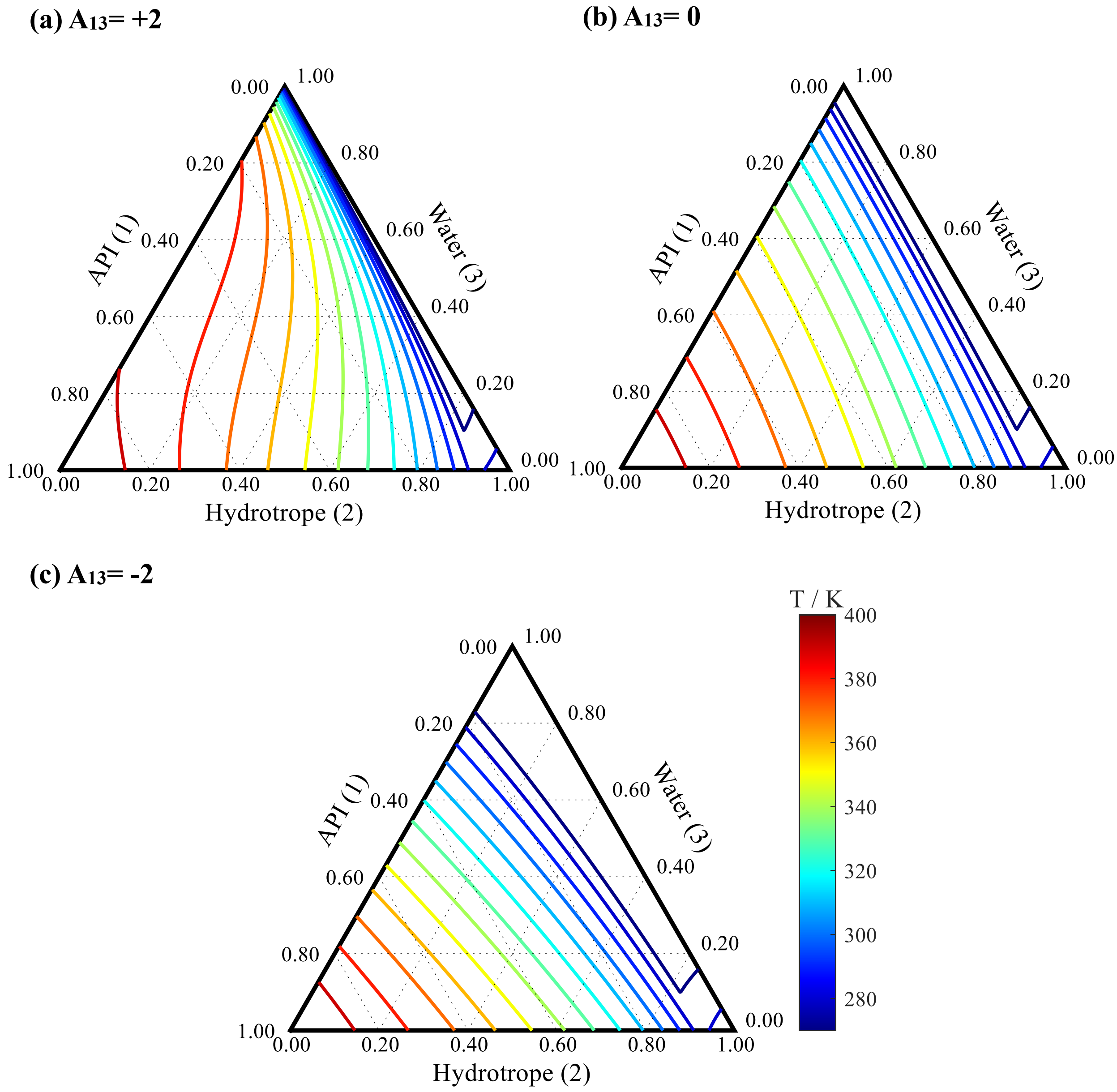

Figure S1. The SLE diagram of a hypothetical ternary API (1)/hydrotrope (2)/water (3) system. Lines represent the solubility isotherms calculated at different temperatures assuming different values of the API-water binary interaction parameter (A_{13}) and $A_{12} = -0.5$ and $A_{23} = -0.5$.

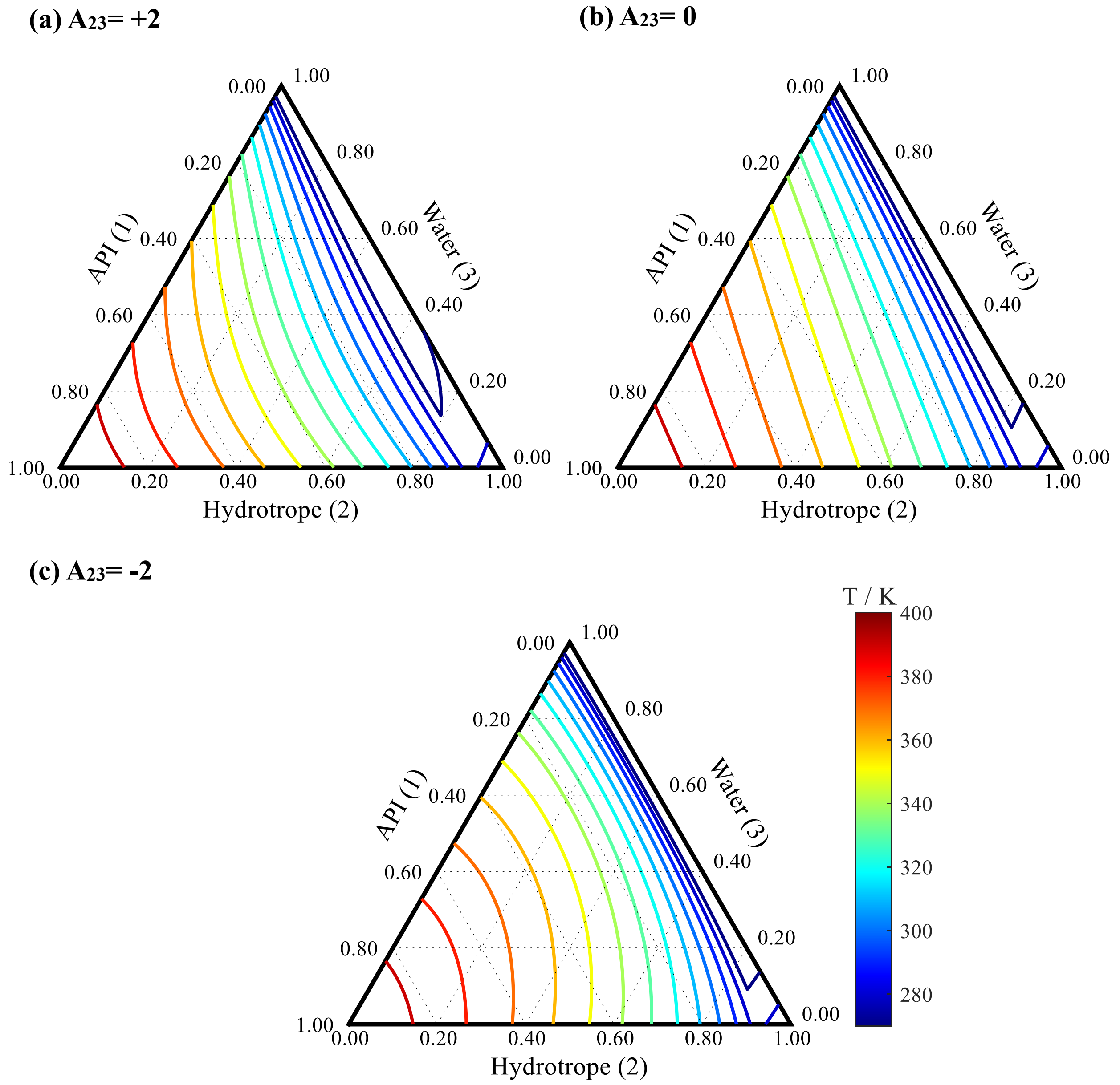

Figure S2. The SLE diagram of a hypothetical ternary API (1)/ hydrotrope (2)/water (3) system. Lines represent the solubility isotherms calculated at different temperatures assuming different values of the hydrotrope -water binary interaction parameter (A_{23}) and $A_{12} = -0.5$ and $A_{13} = +0.5$.

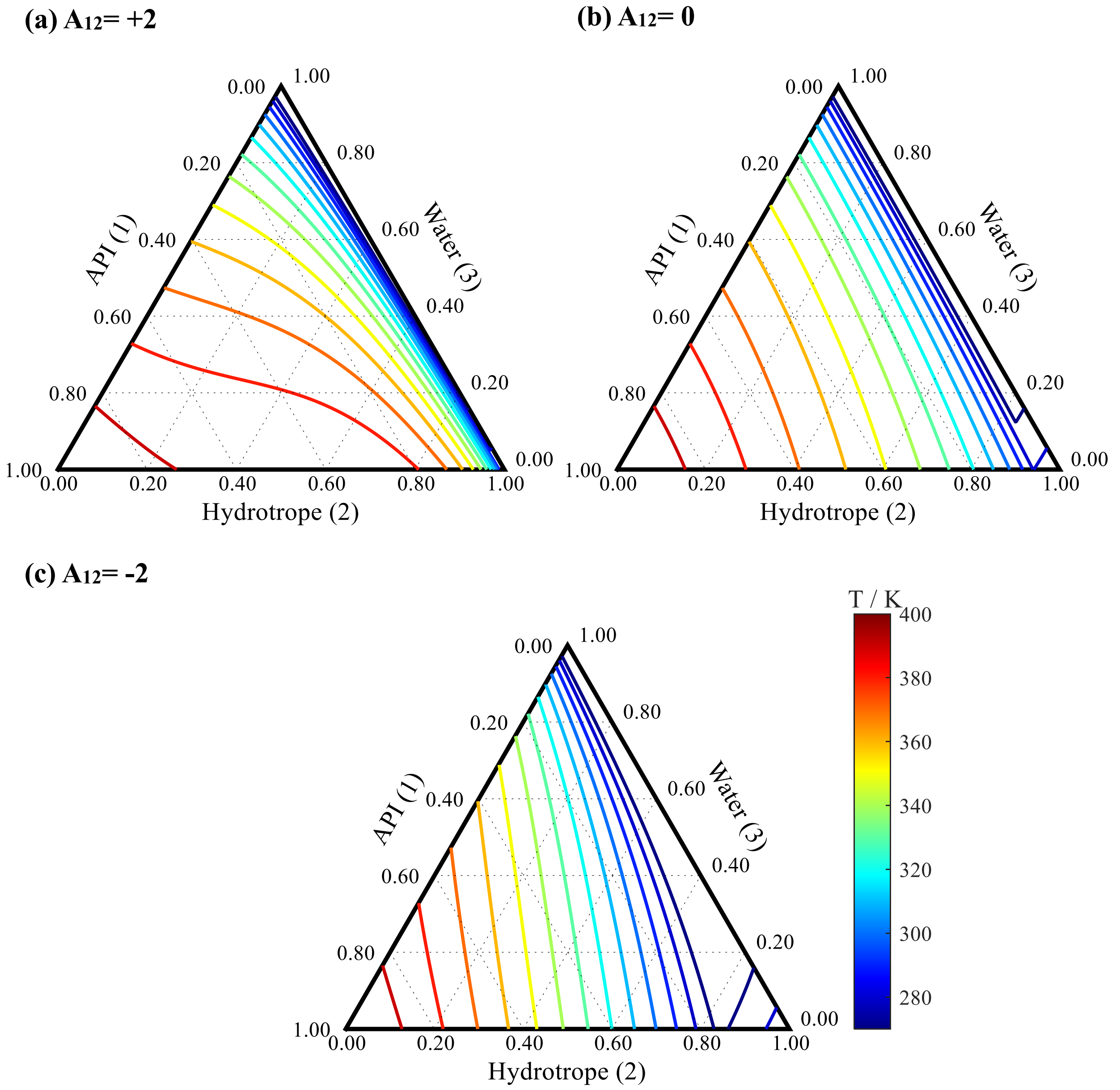

Figure S3. The SLE diagram of a hypothetical ternary API (1)/ hydrotrope (2)/water (3) system. Lines represent the solubility isotherms calculated at different temperatures assuming different values of the API- hydrotrope binary interaction parameter (A_{12}) and $A_{23} = -0.5$ and $A_{13} = +0.5$.

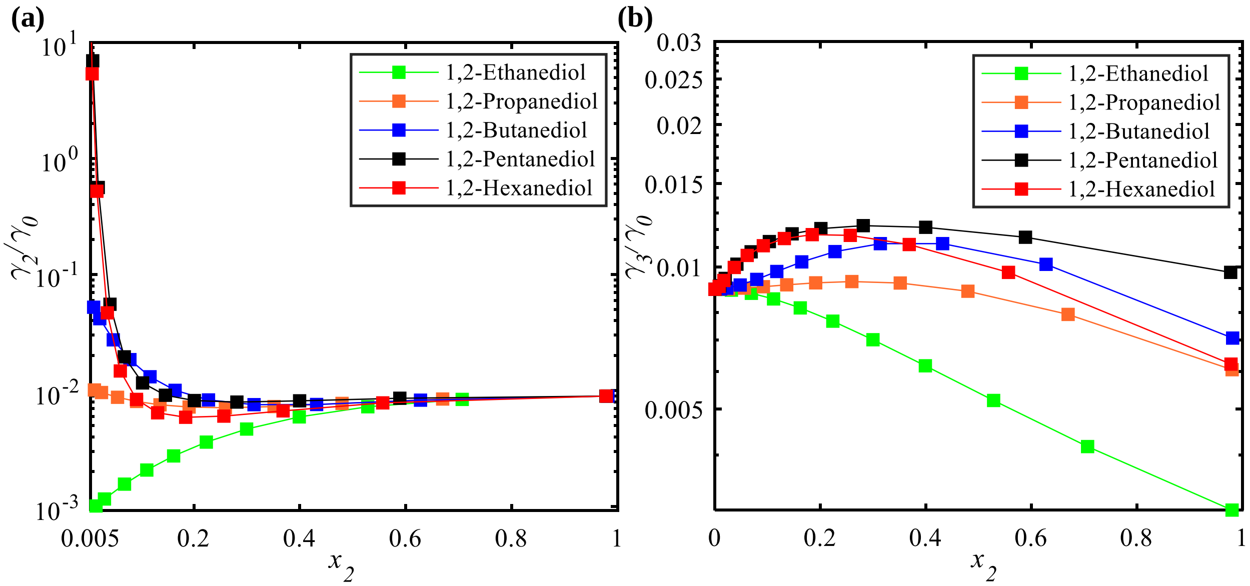

Figure S4. (a) (γ_2/γ_0) calculated along the solubility isotherm for syringic acid (1)/1,2-alkanediols (2)/water (3) system at 303.2 K. (b) (γ_3/γ_0) calculated along the solubility isotherm at 303.2 K for the same systems. In Figures S4 (a) and S4 (b), the squares and the lines were calculated with the NRTL model.

Table S1. Summary of the interaction parameters used for the ternary API (1)/hydrotrope (2)/water (3) system in different sections of the study.

Section	API-hydrotrope interactions (A_{12})	Hydrotrope-water interactions (A_{23})	API-water interactions (A_{13})
3.1.1	−0.5	−0.5	−2 to +2
3.1.2	−0.5	−2 to +2.8	+0.5
3.1.3	−2 to +2	−0.5	+0.5

Table S2. Molecular structures of syringic acid and the studied 1,2-alkanediols.

1,2-Ethanediol 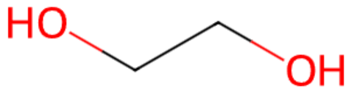	1,2-Propanediol 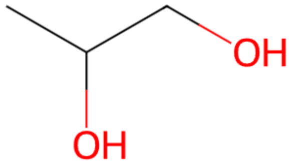
1,2-Butanediol 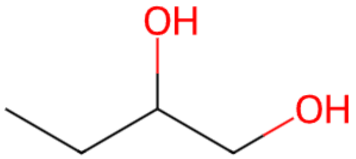	1,2-Pentanediol 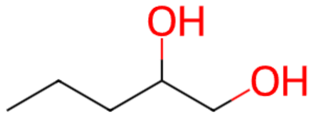
1,2-Hexanediol 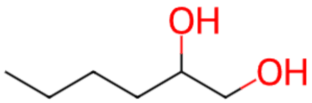	Syringic acid 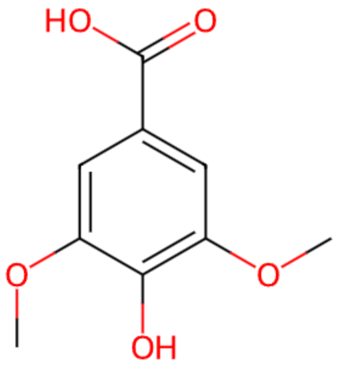
